# Systematic Design of Phononic Band Gap Crystals for Elastic Waves at the Specified Target Frequency via Topology Optimization

**DOI:** 10.3390/ma19030581

**Published:** 2026-02-02

**Authors:** Jingjie He, Zhiyuan Jia, Yuhao Bao, Xiaopeng Zhang

**Affiliations:** 1 Marine Engineering College, Dalian Maritime University, Dalian 116026, China; jingjiehe@dlmu.edu.cn; 2 State Key Laboratory of Structural Analysis, Optimization and CAE Software for Industrial Equipment, Dalian University of Technology, Dalian 116024, China; baoyuhao@mail.dlut.edu.cn (Y.B.); zhangxiaopeng@dlut.edu.cn (X.Z.)

**Keywords:** topology optimization, microstructure design, phononic crystal, specified target frequency

## Abstract

Phononic band gap crystals are characterized by periodic scatterers embedded within a matrix, which enable precise modulation of acoustic or elastic waves. Conventional optimization prioritizes bandwidth maximization, yet practical engineering often requires band gaps at specified frequencies. This requirement creates a significant design challenge. To this end, we develop a topology optimization strategy capable of maximizing elastic wave band gaps around prescribed target frequencies. The approach utilizes Material-Field Series Expansion (MFSE) for unit cell representation and a gradient-free Kriging-based algorithm to tackle the complex optimization problems. This strategy is systematically applied to optimize the band gaps of out-of-plane, in-plane, and complete wave modes, and is further extended to more complex scenarios involving dual-target frequencies. A variety of numerical results demonstrate the method’s effectiveness in engineering phononic crystals for bespoke frequency specifications.

## 1. Introduction

Comprising a periodic array of scatterers embedded within a host matrix, phononic crystals (PnCs) act as functional composite capable of controlling wave propagation [1,2,3,4], which exhibit frequency band gaps in which the transmission of acoustic or elastic waves is completely prohibited [5,6,7]. These properties render PnCs important for a broad spectrum of applications, including noise isolation [2,8], vibration suppression [9,10,11,12], acoustic filtering [13,14,15], defect-based energy harvesting [16,17,18], and waveguiding [19,20,21]. For engineering designs, PnC microstructures can be optimized to deliver either broad band gaps or band gaps centered at specified target frequencies in order to meet performance requirements.

The dispersion behavior of PnCs is strongly dependent on the material distribution of the unit cells [22,23,24,25,26]. As a result, substantial research efforts have been devoted to the systematic design and optimization of unit cell configurations [27,28,29,30,31,32], with a primary focus on structures capable of yielding wide band gaps [33,34,35]. For instance, Sigmund and Jensen [22] employed a gradient-based topology optimization technique to maximize the bandwidth between adjacent bands, while Dong et al. [36] designed the material distribution of the unit cell to optimize band gap width by using genetic algorithms. Additionally, Li et al. [37] reported a topology optimization method to effectively design phononic band gap crystals. Investigations have also extended to multi-material [38,39,40,41] and three-dimensional (3D) PnCs [42,43,44].

Conventional optimization formulations typically focus on maximizing the relative band gap width (gap-to-midgap ratio) between adjacent bands [45,46,47,48,49,50,51,52]. It relies on the scaling law, which implies an inverse proportionality between band gap frequencies and the lattice constant [53,54,55]. However, exclusive reliance on this scaling property presents significant practical limitations [21,56,57]. The fabrication of high-frequency phononic band gap crystal faces manufacturing challenges [17,28]. Moreover, manipulating the interplay between out-of-plane and in-plane wave modes to achieve complete band gaps at high frequencies remains difficult, as it is heavily dependent on the band order [20,56]. Therefore, shifting the optimization objective from maximizing relative width to maximizing the band gap at a predetermined target frequency is more aligned with practical engineering demands.

Despite this necessity, the literature regarding the maximization of band gaps at specified target frequencies remains sparse. Wu et al. [58] proposed a method to define band gap ranges but did not focus on their maximization. For band gap optimization at specified frequencies, Yi et al. [59] maximized the gap between a predefined pair of band orders (e.g., between the third and fourth bands) by constraining its center frequency to match the target. A key feature of their approach was that the optimization was performed for the gap between two predefined band orders. This may pose challenges during the initial design or when the target frequency falls within an unknown band order. In contrast, our strategy does not require predefining the band orders. It can automatically search for and open the widest possible band gap around a given target frequency regardless of the specific band orders involved. Gómez-Silva et al. [60] proposed a topology optimization method for designing PnC microstructures to open band gaps around a target frequency. Their method employed the Bidirectional Evolutionary Structural Optimization (BESO) algorithm with a bi-material interpolation scheme and utilized a small number of eigen frequencies closest to the target frequency in the objective function. It could autonomously determine the optimal material volume fraction during the optimization process. More recently, Wu et al. [61] proposed a novel gradient-based topology optimization method to maximize band gaps at a target frequency. Their approach introduced a distance description function and an ipsilateral frequency constraint, and was validated experimentally. Their work provided an elegant mathematical solution within the gradient-based optimization framework for precise band gap targeting. However, these methods are predominantly limited to single target frequency and in-plane wave mode optimization.

While progress has been made in target frequency optimization, a systematic and versatile design strategy for different wave modes and multiple target frequencies remains a significant challenge. To address this limitation, we introduce the Kriging-based material-field series expansion (KG-MFSE) algorithm [62] derived from the MFSE model [63] to maximize band gaps at specified target frequencies. Our method is systematically implemented for out-of-plane, in-plane, and complete wave modes, and supports both single and dual target frequencies. By providing strong global search capability and limiting convergence to local optima [21,26,35,62,64], the gradient-free strategy demonstrates exceptional capability in precisely handling diverse design scenarios. The optimized PnC microstructures exhibit wide band gaps centered at the specified frequencies, providing compelling numerical evidence of the proposed method’s efficacy. To provide a clearer comparison between the present work and existing studies, Table 1 details the key characteristics of the related optimization strategies. As summarized in Table 1, the present work requires only the target frequency as a preset condition. Its gradient-free KG-MFSE algorithm establishes a unified strategy for the integrated design of single- or dual-target frequency band gaps across all wave modes (in-plane, out-of-plane, and complete). This capability represents the principal advantage of our strategy.

The remainder of this paper is organized as follows. Section 2 introduces the analytical methods for calculating the band structures of PnCs, followed by a detailed description of the objective function formulation and the series expansion method for design variables. Section 3 presents the systematic designs for optimizing phononic band gap crystals at specified target frequencies, covering in-plane, out-of-plane, and complete wave modes, as well as dual-target frequency problems. The accuracy and validity of the optimization are verified through frequency response calculations. Section 4 concludes this work.

## 2. Theoretical Framework and Optimization Formulation

Due to its versatility, the finite element method is widely utilized for computing the dispersion relations of PnCs. This section begins by outlining the discretization approach employed for band structure analysis by the finite element method. Following this, a topology optimization strategy incorporating signed minimal distance measures is introduced. Moreover, the KG-MFSE algorithm is leveraged to characterize and design phononic crystal configurations.

### 2.1. Analysis of Band Gap Behavior

When elastic waves propagate within PnCs, their behavior is governed by the following equation:

(1) $$\rho \left(r\right)\frac{\partial^{2}U}{\partial t^{2}}=\nabla \left[\lambda \left(r\right)+2\mu \left(r\right)\left(\nabla \cdot U\right)\right]-\nabla \times \left[\mu \left(r\right)\nabla \times U\right]$$

where $\rho \left(r\right)$ represents the mass density, $U={\left[u,v,w\right]}^{T}$ is the displacement vector, and $\lambda \left(r\right)$ and $\mu \left(r\right)$ denote the Lame’s parameters. For a two-dimensional (2D) phononic crystal, elastic wave motion confined to the x-y plane may be classified into a single out-of-plane shear mode and a pair of in-plane modes arising from the coupling between longitudinal and transverse components. Assuming invariance of the wave field along the z-direction, such that ∂U/∂z=0, the position vector reduces to $r\left(x,y\right)$. Under these conditions, the governing equations associated with the in-plane longitudinal and transverse wave modes are given as follows:

(2) $$\rho \left(r\right)\frac{\partial^{2}u}{\partial t^{2}}=\frac{\partial }{\partial x}\left[\left(\lambda \left(r\right)+2\mu \left(r\right)\right)\frac{\partial u}{\partial x}+\lambda \left(r\right)\frac{\partial v}{\partial y}\right]+\frac{\partial }{\partial y}\left[\mu \left(r\right)\left(\frac{\partial u}{\partial y}+\frac{\partial v}{\partial x}\right)\right]$$

(3) $$\rho \left(r\right)\frac{\partial^{2}v}{\partial t^{2}}=\frac{\partial }{\partial x}\left[\mu \left(r\right)\left(\frac{\partial v}{\partial x}+\frac{\partial u}{\partial y}\right)\right]+\frac{\partial }{\partial y}\left[\left(\lambda \left(r\right)+2\mu \left(r\right)\right)\frac{\partial v}{\partial y}+\lambda \left(r\right)\frac{\partial u}{\partial x}\right]$$

By comparison, the governing equation associated with the out-of-plane shear wave mode is given by

(4) $$\rho \left(r\right)\frac{\partial^{2}w}{\partial t^{2}}=\frac{\partial }{\partial x}\left[\mu \left(r\right)\frac{\partial w}{\partial x}\right]+\frac{\partial }{\partial y}\left[\mu \left(r\right)\frac{\partial w}{\partial y}\right]$$

To deal with infinite periodic structures, the Floquet–Bloch theorem is introduced. It states that the displacement field can be written as the product of a periodic function and a plane wave phase factor. The periodic part carries the material’s periodicity, while the plane wave factor contains the wave vector and frequency information. This transformation restricts the original problem to solving within a single representative unit cell, and limits the wave vector to the first Brillouin zone in reciprocal space. The Floquet–Bloch theorem reads

(5) $$U\left(r,k\right)=U_{k}\left(r\right)e^{i\left(\omega t+k\cdot r\right)}$$

where $U_{k}\left(r\right)$ represents the periodic displacement field, $k=\left(k_{x},k_{y}\right)$ is the Bloch wave vector, and ω is the corresponding angular frequency (ω=2πf). Owing to periodicity of phononic crystal, material properties are periodic with respect to the lattice translation vector a, i.e., $\lambda \left(r\right)=\lambda \left(r+a\right)$, $\mu \left(r\right)=\mu \left(r+a\right)$, and $\rho \left(r\right)=\rho \left(r+a\right)$. The 2D phononic crystals examined in this work are organized in a square lattice with lattice constant a.

In the analysis of elastic wave propagation in periodic media, the finite element method has become the predominant numerical approach due to its strong capability in handling complex geometries and heterogeneous material distributions. The analysis typically begins by extracting a representative unit cell from the infinite periodic structure, followed by discretization of its geometric domain. The unit cell is subdivided into a mesh of interconnected finite elements, with each element assigned specific material properties to accurately represent the spatial variation of materials within the periodic medium. Through this discretization process, the continuous dynamic problem is transformed into a set of algebraic equations governing the nodal displacement fields within the unit cell. By substituting Equation (5) into the governing equations, the problem is transformed into two decoupled standard eigenvalue problems, which describe the propagation of in-plane and out-of-plane wave modes. Mathematically, the eigenvalue problems corresponding to the in-plane and out-of-plane wave modes are formulated as

(6) $$\left(K-\omega^{2}M\right)u=0$$

where u denotes the global displacement vector. The explicit forms of the stiffness matrix K and mass matrix M can be found in the cited reference [37]. By solving the eigenvalue problem, the dispersion relation of the periodic structure is obtained, thereby providing a quantitative basis for the analysis of band gaps and wave propagation characteristics.

**Figure 1:**
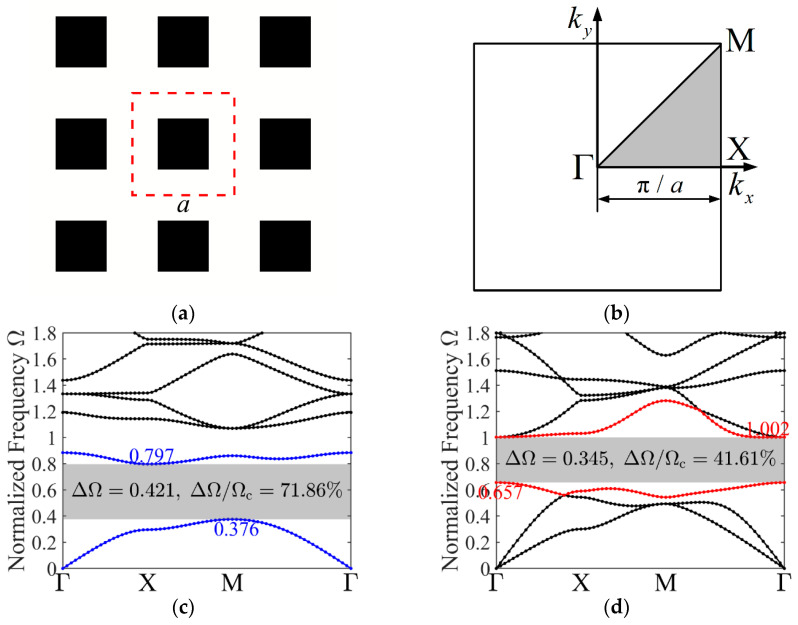
A 2D phononic crystal and band structures. (**a**) The unit cells (3 × 3); (**b**) IBZ; (**c**) the out-of-plane mode band structure; (**d**) the in-plane mode band structure.

Figure 1a depicts a 2D phononic crystal arranged in a periodic square lattice, composed of inclusions (black) and a matrix (white). While the presented designs are applicable to infinite periodic structures, they are visualized here as a 3 × 3 array, and the representative unit cell is enclosed within red dashed lines. This finite assembly allows for a clearer illustration of the spatial arrangement and geometry of the presented design. The geometry of this structure is defined by the lattice constant a. The side length of the square inclusion is 0.5a. To mitigate scaling dependency, the frequency is normalized as $\Omega \left(\Omega =\omega a/\left(2\pi c_{tm}\right)\right)$, with $c_{tm}$ denoting the transverse wave velocity of the matrix. Figure 1b depicts the Irreducible Brillouin Zone (IBZ). The boundary follows Γ-X-M-Γ, with the wave vectors $k=\left(k_{x},k_{y}\right)$ discretized into eleven equally spaced points along each segment. It starts from $\Gamma \left(0,0\right)$ to $Χ\left(\pi /a,0\right)$, then to $Μ\left(\pi /a,\pi /a\right)$, and finally returns to Γ. Consequently, the band structure, or dispersion curve $\left(k-\Omega \right)$, is derived. The resulting band structures for in-plane and out-of-plane wave modes are shown in Figure 1c and Figure 1d, respectively. The gray regions in these plots correspond to the band gaps. Their upper limit $\Omega_{u}$ and lower limit $\Omega_{l}$ are shown, allowing the center frequency to be defined as $\Omega_{c}\;\left(\Omega_{c}=\left(\Omega_{u}+\Omega_{l}\right)/2\right)$. Furthermore, the absolute band gap width $∆\Omega \left(∆\Omega =\Omega_{u}-\Omega_{l}\right)$ and the relative width of the band gap (gap-to-midgap ratio) $∆\Omega /\Omega_{c}$ are also marked.

### 2.2. Formulation of the Topology Optimization Problem

The present study seeks to determine the optimized phononic crystal configurations that yields the largest band gap around a given target frequency $\Omega^{*}$. However, band tracking, namely the precise identification of the corresponding band orders, is particularly challenging because mode switching and band crossings can arise throughout the iterative optimization process. To circumvent this difficulty, the signed minimal distance measure, denoted by $\Delta_{m}$, is adopted. This parameter indicates the minimal separation between $\Omega^{*}$ and the adjacent band frequencies, $\Omega_{m}\left(k\right)$ and $\Omega_{m+1}\left(k\right)$, across the entire IBZ. Mathematically, $\Delta_{m}$ is formulated as

(7) $$\begin{array}{c} \Delta_{m}\left(\Omega^{*}\right)=min\left\{\Omega^{*}-\underset{i=1,2,\cdots ,n_{k}}{max}\Omega_{m}\left(k_{i}\right),\underset{i=1,2,\cdots ,n_{k}}{min}\Omega_{m+1}\left(k_{i}\right)-\Omega^{*}\right\}\;\;\;\\ m=1,\;2,\;3,\cdots ,M \end{array}$$

where $n_{k}$ corresponds to the total number of sampled wave vectors k along the path, and M determines the maximum band order included in the band structure analysis. Specifically, M is set to M=30 for out-of-plane and M=45 for in-plane and complete wave modes.

**Figure 2:**
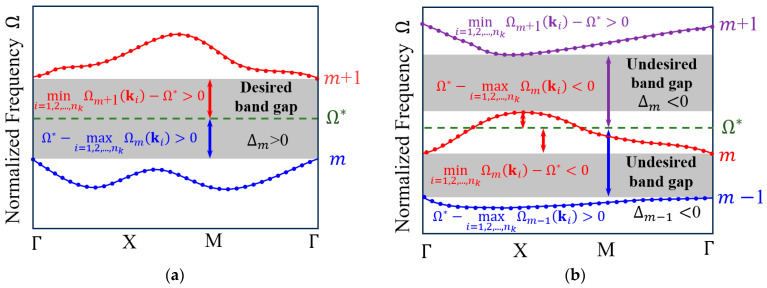
Schematic representations of the signed minimal distance measures. (**a**) A case with $\Delta_{m}>0$; (**b**) a case with $\Delta_{m}<0$ and $\Delta_{m-1}<0$.

The signed minimal distance measure $\Delta_{m}$ is shown in Figure 2. As illustrated in Figure 2a, a positive $\Delta_{m}$ is obtained when a band gap, located between two adjacent bands (m and m+1), encompasses $\Omega^{*}$. Conversely, in Figure 2b, a non-positive value indicates the absence of a band gap near $\Omega^{*}$. $\Delta_{m}$ and $\Delta_{m-1}$ can take negative values if the target frequency $\Omega^{*}$ lies outside the band gap. When $\Omega^{*}$ is positioned equidistantly between two consecutive bands, $\Delta_{m}$ takes the large value of minimal distance. In other words, it evaluates the deviation of the band gap boundaries from the target frequency while simultaneously guiding the optimization to center the band gap around $\Omega^{*}$.

Throughout the optimization process, potential variations in the phononic crystal band orders necessitate the calculation of the signed minimal distances between the $\Omega^{*}$ and nearby bands. The objective function is formulated by

(8) $$\delta \left(\Omega^{*}\right)=\max\left\{\Delta_{1}\left(\Omega^{*}\right),\Delta_{2}\left(\Omega^{*}\right),\Delta_{3}\left(\Omega^{*}\right),\cdots ,\Delta_{M}\left(\Omega^{*}\right)\right\}$$

Gradient-based topology optimization typically requires the computation of objective function sensitivities to iteratively update a large set of design variables. In the present work, however, the discontinuous and intricate form of the objective function renders sensitivity evaluation challenging. Approximate approaches such as aggregation functions or finite difference schemes are available, the gradient-based topology optimization process exhibits pronounced instability and susceptibility to local optima due to the multi-peak characteristics of the objective function. To overcome these difficulties, this study employs the KG-MFSE algorithm [62]. Being gradient-free, KG-MFSE has demonstrated effectiveness in optimizing comparable objective functions in photonic crystals [65] and PnCs [15,66].

The optimization model is constructed via the MFSE method [63] within the KG-MFSE framework. The method facilitates the representation of complex material topologies by a small number of design variables, typically around 50 per unit cell, while remaining unaffected by the mesh resolution.

The observation points $s_{i}\left(i=1,2,\cdots ,N_{P}\right)$ are evenly distributed in the design domain $S_{des}$, and the material distribution is represented by the field function $\varphi \left(s\right)\in \left[-1,1\right]$, where $s\in S_{des}$. Spatial correlations among different locations (e.g., $s_{m}$ and $s_{n}$) are taken into account, with $l_{c}$ specifying the correlation length. This field is expanded in terms of independent design variables η, ensuring that the resulting values $\varphi \left(s,\eta \right)$ remain within the prescribed bounds $\varphi \in \left[-1,1\right]$ at all observation points.

The unit cell of the phononic crystal is partitioned into *N*_ele_ finite elements, and the material-field function defines the relative density of each element as follows:

(9) $$x_{e}\left(\eta \right)=\frac{1+\varphi \left(s_{e},\eta \right)}{2},\left(e=1,2,\cdots ,N_{\mathrm{ele}}\right)$$

where $\eta =\left\{\eta_{1},\eta_{2},\cdots ,\eta_{N}\right\}$ denotes the set of design variables, which parameterize the material field in a reduced transformation space. In this work, the reduced space dimension is set to N=50. The relative density $x_{e}$ of each finite element controls its material characteristics, and $s_{e}$ denotes the element centroid position.

KG-MFSE demonstrates both strong capability for global optimization and gradient-free characteristics. This is important in the complex material design problems of phononic crystal unit cells, where a number of local optima are present and the optimization strongly depends on the initial design. Readers interested in the derivation and implementation of KG-MFSE are referred to reference [62].

Based on the preceding discussions, the optimization problem is formulated as

(10) $$\begin{array}{c} \underset{\eta }{\min}:-\delta \left(\Omega^{*}\right)\\ s.t.:\left(K-\omega^{2}M\right)u=0\\ \varphi \left(s_{i},\eta \right)\in \left[-1,1\right],\;\left(i=1,2,\cdots ,N_{P}\right) \end{array}$$

**Figure 3:**
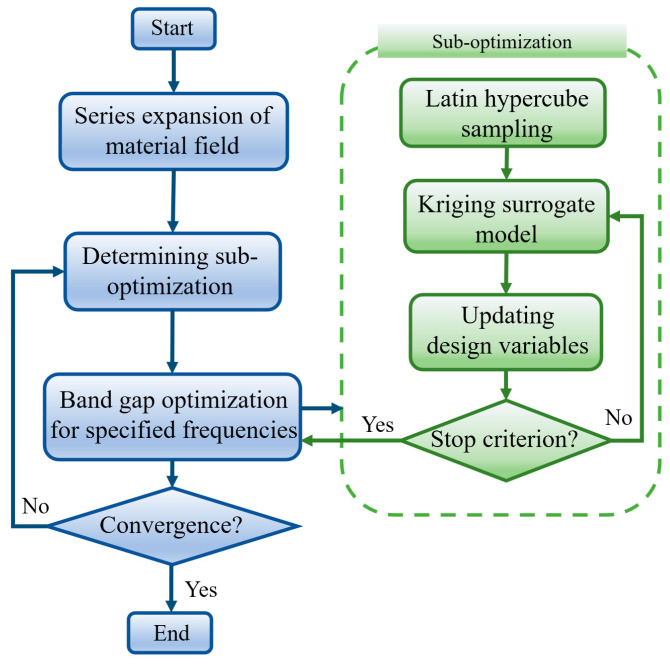
Flowchart of the proposed topology optimization strategy.

The following material interpolation schemes are employed to evaluate the material properties of the e-th element:

(11) $$\rho_{e}\left(\eta \right)=\rho_{1}+{\left(x_{e}\left(\eta \right)\right)}^{p_{\rho }}\left(\rho_{2}-\rho_{1}\right)$$

(12) $$\lambda_{e}\left(\eta \right)=\lambda_{1}+{\left(x_{e}\left(\eta \right)\right)}^{p_{\lambda }}\left(\lambda_{2}-\lambda_{1}\right)$$

(13) $$\mu_{e}\left(\eta \right)=\rho_{1}+{\left(x_{e}\left(\eta \right)\right)}^{p_{\mu }}\left(\mu_{2}-\mu_{1}\right)$$

where the subscripts ‘1’ and ‘2’ correspondingly represent Material 1 and Material 2. The penalization exponents applied to the density interpolation of the mass density, Lame’s first parameter, and shear modulus are denoted by $p_{\rho }$, $p_{\lambda }$, and $p_{\mu }$, and are assigned values of 1, 3, and 3, respectively. The proposed topology optimization strategy is summarized in the flowchart shown in Figure 3.

## 3. Numerical Investigations and Validation

The subsequent numerical findings are obtained via the proposed topology optimization strategy applied to a square unit cell with lattice constant a. The unit cell possesses $C_{4v}$ symmetry. The unit cell is divided into an 80 × 80 finite element mesh. Considering symmetry, one-quarter of the unit cell is selected as the design domain, corresponding to a 40 × 40 mesh. The design domain contains $N_{P}=1600$ uniformly distributed observation points. The topology optimization strategy proposed in this study demonstrates broad adaptability and can be applied to the unit cells with different symmetries or three-dimensional geometries through appropriate modifications. However, the extensive finite element analysis required for three-dimensional cases entails considerable computational costs. This is a challenge that needs to be addressed in future research.

By employing the normalized frequency Ω, the optimization results exhibit independence from the specific choice of the lattice constant a, and a=0.1 m is used in this work. $l_{c}$ is correlation length of material field, and $l_{c}=0.01\;m$, corresponding to 20% of the design domain width. Research on two-phase solid–solid PnCs typically prioritizes materials with contrasting properties to promote strong interfacial wave scattering, a mechanism that favors the generation of band gaps. Accordingly, epoxy matrix (Material 1, shown in white) and steel inclusion (Material 2, shown in black) are selected in this study. Beyond their substantial impedance mismatch, these materials are cost effective and exhibit excellent manufacturability, rendering them ideal for practical engineering applications. The material properties are adopted from reference [1] as follows: for Epoxy, $\rho_{1}=1180\;kg\cdot m^{-3}$,$\;\lambda_{1}=4.44$ GPa and $\mu_{1}=1.59$ GPa; for Steel, $\rho_{2}=7800$ $kg\cdot m^{-3}$, $\lambda_{2}=113.47$ GPa and $\mu_{2}=80.87$ GPa.

The algorithm is executed over 15 sub-optimization stages, totaling 2715 steps. Owing to the use of Latin Hypercube Sampling (LHS), the KG-MFSE method generally requires more finite element evaluations than conventional gradient-based optimization methods. However, this increased computational cost enables a stronger capability for global exploration. Additionally, LHS eliminates the need for a specific starting design, so the initial configuration can be set freely.

### 3.1. Optimized Designs for Out-of-Plane Wave Mode

**Figure 4:**
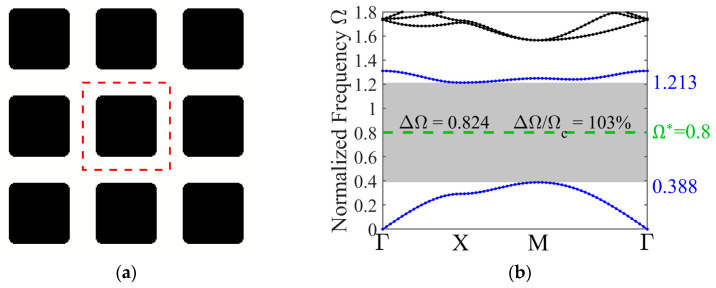
(**a**) Characterization of the optimized 3 × 3 unit cell design; (**b**) the out-of-plane mode band structure.

The initial investigation addresses the PnC microstructure designs for out-of-plane wave modes. The target normalized frequencies in this study are set to 0.8, 2, and 3. It should be noted that these values are user-definable. Here, the three target frequencies are selected arbitrarily to demonstrate the applicability and flexibility of the proposed strategy under varying frequency conditions. While the specified target normalized frequency is set to $\Omega^{*}=0.8$, the optimized configuration of the phononic crystal unit cell is shown in Figure 4a.

A 3 × 3 unit cell arrangement is presented and the representative unit cell is enclosed within the red dashed grid lines. The optimized result reveals a rounded-rectangle inclusion with a highly discernible interface.

Figure 4b illustrates the out-of-plane band structure for the optimized design, where the prescribed target frequency is indicated by the green dashed horizontal line. The band gap closest to the target frequency $\Omega^{*}=0.8$ is highlighted by a gray overlay. The two bands adjacent to the band gap are plotted as blue lines, with their specific values labeled. For out-of-plane wave mode, it is evident that the specified target frequency aligns closely with the central frequency of the resulting band gap. Moreover, a large absolute and relative band gap width is achieved, both of which are indicated in the figure. The relative band gap width reaches 0.824, and the absolute width attains 103%, demonstrating the effectiveness of the proposed method.

To validate the band gap characteristics of the optimized configuration, frequency response simulations are conducted using a conceptual optimized model of the unit cell. Specifically, the out-of-plane wave propagation is modeled by the COMSOL Multiphysics 5.3, leveraging the Pressure Acoustics module to simulate the frequency response by using 1/μ for the material density and $\sqrt{\mu /\rho }$ for the longitudinal wave velocity in the frequency domain calculations [56,67].

**Figure 5:**
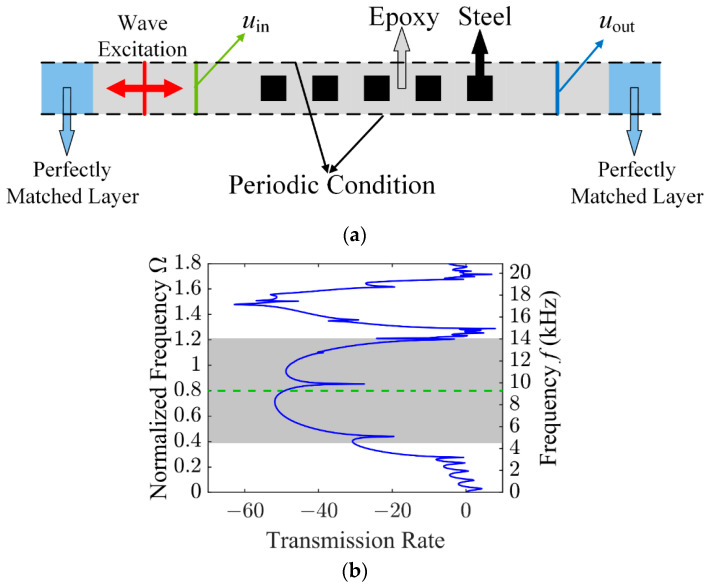
(**a**) Configuration of the computational model for frequency response analysis; (**b**) corresponding transmission spectrum.

The frequency response analysis is performed using a 5 × 1 configuration of the optimized unit cell, as shown in Figure 5a. Vertical periodicity is enforced via periodic boundary conditions applied to the top and bottom surfaces, effectively modeling an infinite lattice. A sinusoidal excitation is imposed at the left end, with the excitation location indicated by the red line in the figure, and a sweep over a range of frequencies is conducted. The input and output positions of interest are marked by the green and blue lines, respectively. To mitigate the influence of reflected waves, perfectly matched layers are placed at both ends of the structure. The transmission rate (TR) is defined as follows:

(14) $$\mathrm{TR}=10\times \log_{10}\left(u_{out}/u_{in}\right)$$

where $u_{out}$ and $u_{in}$ correspond to the displacements at the emission point and the reception point, respectively. The transmission spectrum is obtained by sweeping the frequency from 0 to 1.8, which is illustrated in Figure 5b. In the transmission spectrum, the band gap region is shaded in gray, and the specified target frequency is marked by the green dashed line. As observed from the transmission curve, the elastic wave transmission undergoes significant attenuation within the band gap region, thereby confirming the effectiveness of the optimized design.

**Figure 6:**
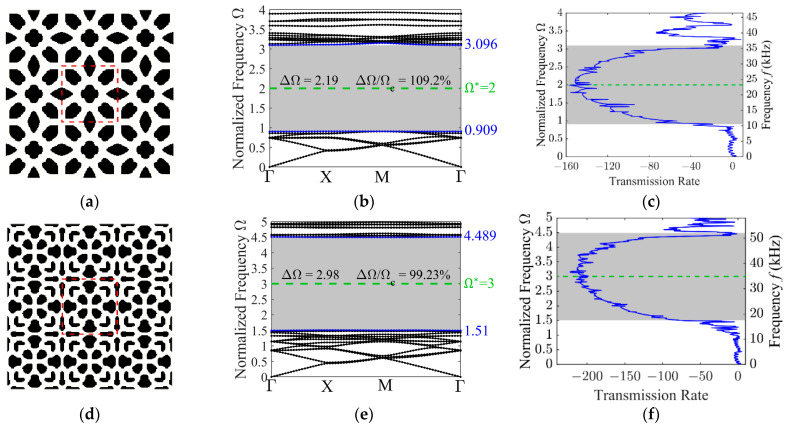
Characterization of the optimized 3 × 3 unit cell designs for various target frequencies, along with their corresponding band structures and transmission spectra. The optimized configurations: (**a**) $\Omega^{*}=2$; (**d**) $\Omega^{*}=3$; the out-of-plane mode band structures: (**b**) $\Omega^{*}=2$; (**e**) $\Omega^{*}=3$; the transmission spectra: (**c**) $\Omega^{*}=2$; (**f**) $\Omega^{*}=3$.

Next, optimized PnC unit cell configurations corresponding to different target frequencies are presented. For the target frequency of 2, the optimized topology shown in Figure 6a manifests increased complexity, with the inclusions exhibiting a more spatially dispersed configuration than those in Figure 4a. Such intricacy is attributable to the engagement of higher order modes as the frequency target shifts upward. Both the corresponding band structures in Figure 6b and the transmission spectrum in Figure 6c demonstrate successful band gap maximization around the prescribed target frequency (green dashed line). Notably, the target frequency is closely aligning with the center of the resulting band gap, yielding an absolute bandwidth of 2.19 and a relative width of 109.2%.

A similar observation can be made for the case with a target frequency of 3. Figure 6d–f present the optimized configuration, the associated band structure, and the transmission spectrum. In this case as well, a pronounced band gap is successfully formed around the specified target frequency, whose absolute and relative band gap widths are 2.98 and 99.23%, respectively. This provides further evidence of the effectiveness of the proposed approach.

Departing from prior studies that primarily aimed to maximize the band gap width between adjacent bands of a specific order [45,46,47,48,49,50,51,52], this work focuses on maximizing the band gap width in the vicinity of a target frequency. Crucially, the band gap order is not predetermined but is adaptively determined during optimization. The results demonstrate that the generated band gaps appear around the target frequency, with their mid-gap frequencies closely aligning with it. Consequently, this strategy proves highly advantageous for engineering applications demanding tailored frequency ranges.

### 3.2. Optimized Designs for In-Plane Wave Mode

**Figure 7:**
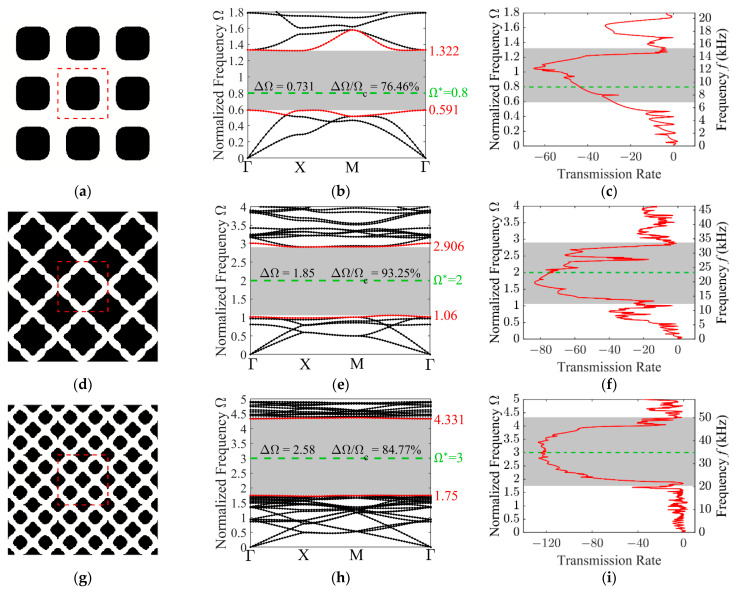
Characterization of the optimized 3 × 3 unit cell designs for various target frequencies, along with their corresponding band structures and transmission spectra. The optimized configurations: (**a**) $\Omega^{*}=0.8$; (**d**) $\Omega^{*}=2$; (**g**) $\Omega^{*}=3$; the in-plane mode band structures: (**b**) $\Omega^{*}=0.8$; (**e**) $\Omega^{*}=2$; (**h**) $\Omega^{*}=2$; the transmission spectra: (**c**) $\Omega^{*}=0.8$; (**f**) $\Omega^{*}=2$; (**i**) $\Omega^{*}=3$.

In this section, the focus shifts to the topology optimization of PnCs specifically tailored for the in-plane wave mode. Due to the inherent coupling between longitudinal and transverse components in elastodynamics, the wave motion and the associated dispersion relations manifest complexity exceeding that of the decoupled out-of-plane mode. Consequently, the corresponding dispersion curves are more intricate. In this case, the target frequencies are chosen to be consistent with those of the out-of-plane mode, namely 0.8, 2, and 3. The optimization aims at tailoring unit cell topologies to achieve maximum band gap widths at the target frequencies. To provide a comprehensive validation, Figure 7 summarizes the resulting optimized unit cell along with their corresponding band structures and transmission spectra for the different target frequencies. The dispersion curves and transmission spectra clearly show that the proposed method effectively realizes maximized band gaps that include the specified target frequencies (marked by the green dashed lines), thereby confirming the validity of the approach. Nevertheless, the presence of longitudinal and transverse waves coupling leads to a more pronounced discrepancy between the target frequency and the band gap center compared with the out-of-plane cases, as shown in Figure 7b.

### 3.3. Optimized Designs for Complete Wave Mode

In this section, topology optimization is performed to maximize complete band gaps around the specified target frequencies. The realization of a complete band gap necessitates the simultaneous evaluation of in-plane and out-of-plane wave modes, thereby ensuring that elastic waves cannot propagate within the band gap range regardless of their incidence direction or polarization state. Nevertheless, optimizing the complete band gap remains challenging, as the appropriate combination of band orders between out-of-plane and in-plane waves can scarcely be determined in advance.

**Figure 8:**
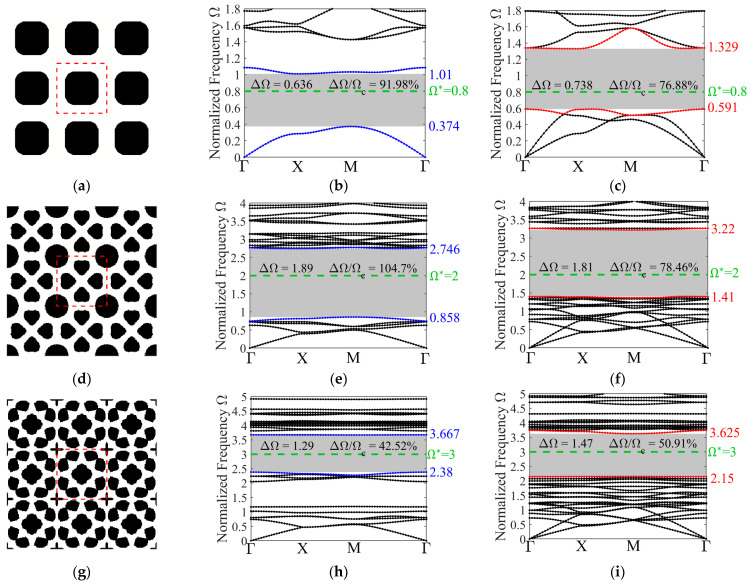
Characterization of the optimized 3 × 3 unit cell designs for various target frequencies, along with their corresponding band structures for out-of-plane and in-plane modes: the optimized configurations: (**a**) $\Omega^{*}=0.8$; (**d**) $\Omega^{*}=2$; (**g**) $\Omega^{*}=3$; the out-of-plane mode band structures: (**b**) $\Omega^{*}=0.8$; (**e**) $\Omega^{*}=2$; (**h**) $\Omega^{*}=2$; the in-plane mode band structures: (**c**) $\Omega^{*}=0.8$; (**f**) $\Omega^{*}=2$; (**i**) $\Omega^{*}=3$.

The target frequencies are again chosen as 0.8, 2, and 3 to ensure consistency. Figure 8 presents the optimized phononic crystal unit cells corresponding to these target frequencies, along with the dispersion curves for the out-of-plane and in-plane modes. These examples demonstrate the successful achievement of the optimization objective. However, it is inevitable that designs incorporating complete band gaps result in narrower band gap widths compared to those for the single mode, and the deviation between the target frequency and the band gap center frequency becomes larger. This is a direct consequence of the heightened complexity within the optimization problem. For instance, in Figure 8b, the absolute and relative band gaps are 0.636 and 91.98%, respectively, both of which are reduced compared with those in Figure 4b.

### 3.4. Optimized Designs for Dual-Target Frequencies

To further illustrate the versatility and efficacy of the proposed strategy, we examine topology optimization subject to two distinct target frequency combinations. For the dual-target-frequency scenario, the optimization formulation is adapted as follows:

(15) $$\begin{array}{c} \underset{\eta }{\min}:\left\{-\delta \left(\Omega_{1}^{*}\right),-\delta \left(\Omega_{2}^{*}\right)\right\}\\ s.t.\;:\;\left(K-\omega^{2}M\right)u=0\\ \varphi \left(s_{i},\eta \right)\in \left[-1,1\right],\;\left(i=1,2,\cdots ,N_{P}\right) \end{array}$$

where $\Omega_{1}^{*}$ and $\Omega_{2}^{*}$ are two different specified target frequencies.

Specifically, the target frequency combinations considered in this section are {0.8, 2}, {0.8, 3}, and {2, 3}. For each combination, we sequentially perform topology optimization for out-of-plane, in-plane, and complete wave modes.

**Figure 9:**
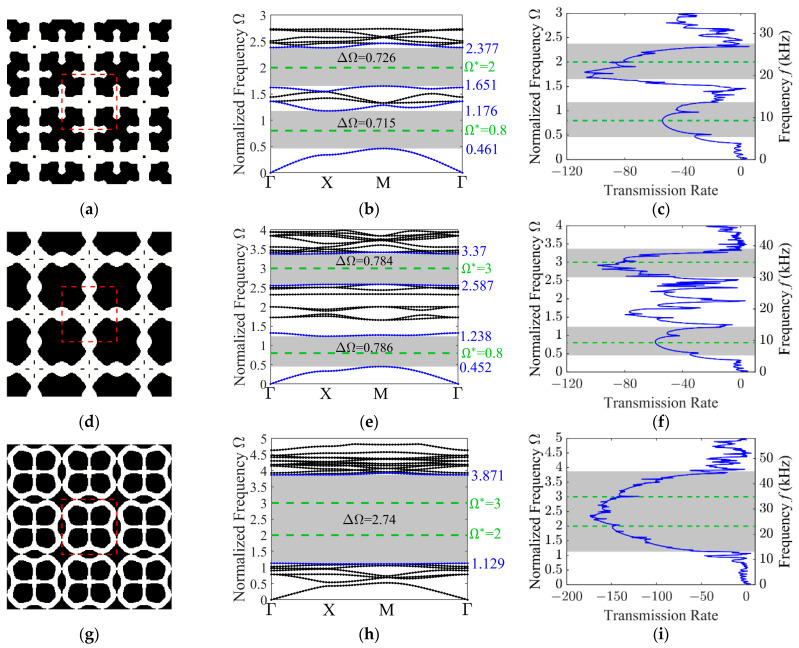
Characterization of the optimized 3 × 3 unit cell designs for various target frequency combinations, along with their corresponding band structures and transmission spectra. The optimized configurations: (**a**) {0.8, 2}; (**d**) {0.8, 3}; (**g**) {2, 3}; the out-of-plane mode band structures: (**b**) {0.8, 2}; (**e**) {0.8, 3}; (**h**) {2, 3}; the transmission spectra: (**c**) {0.8, 2}; (**f**) {0.8, 3}; (**i**) {2, 3}.

First, the designs for out-of-plane modes with dual-target frequencies are presented. Figure 9 illustrates the optimized microstructure designs, along with their corresponding dispersion curves and transmission spectra. In these plots, the target frequencies are marked by green dashed lines.

For the target frequency pair {0.8, 2}, the dispersion curve reveals two independent band gaps with absolute widths of 0.715 and 0.726. Similarly, for the target frequencies {0.8, 3}, two independent band gaps are observed with absolute widths of 0.786 and 0.784. When the target frequencies are {2, 3}, the dispersion curve displays a single broad, independent band gap with an absolute width of 2.74. These results demonstrate that the proposed strategy successfully enables the optimization of out-of-plane wave mode at dual-target frequencies.

**Figure 10:**
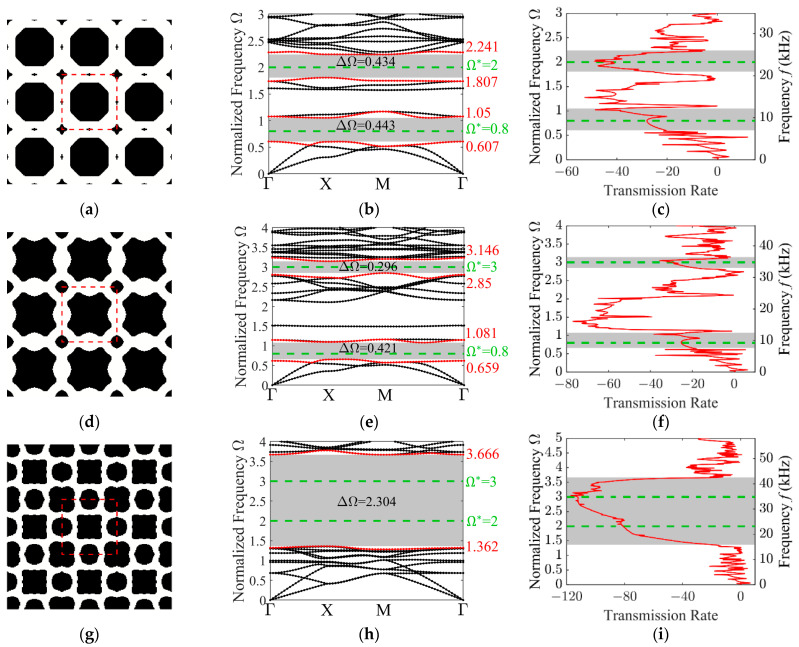
Characterization of the optimized 3 × 3 unit cell designs for various target frequency combinations, along with their corresponding band structures and transmission spectra. The optimized configurations: (**a**) {0.8, 2}; (**d**) {0.8, 3}; (**g**) {2, 3}; the in-plane mode band structures: (**b**) {0.8, 2}; (**e**) {0.8, 3}; (**h**) {2, 3}; the transmission spectra: (**c**) {0.8, 2}; (**f**) {0.8, 3}; (**i**) {2, 3}.

Next, the optimized designs for in-plane mode at dual-target frequencies are presented. Figure 10 illustrates the optimized unit cell designs, along with their corresponding dispersion curves and transmission spectra. The target frequencies are marked by green dashed lines in these plots.

For the target frequency pair {0.8, 2}, the dispersion curve reveals two independent band gaps with absolute widths of 0.443 and 0.434, respectively. Similarly, for the target frequencies {0.8, 3}, two independent band gaps are observed with absolute widths of 0.421 and 0.296. When the target frequencies are set to {2, 3}, the dispersion curves reveal a single isolated band gap with an absolute width of 2.304. Compared with the out-of-plane modes, the coupling effect of elastic waves in the in-plane modes results in more complex dispersion curves and reduced band gap widths.

**Figure 11:**
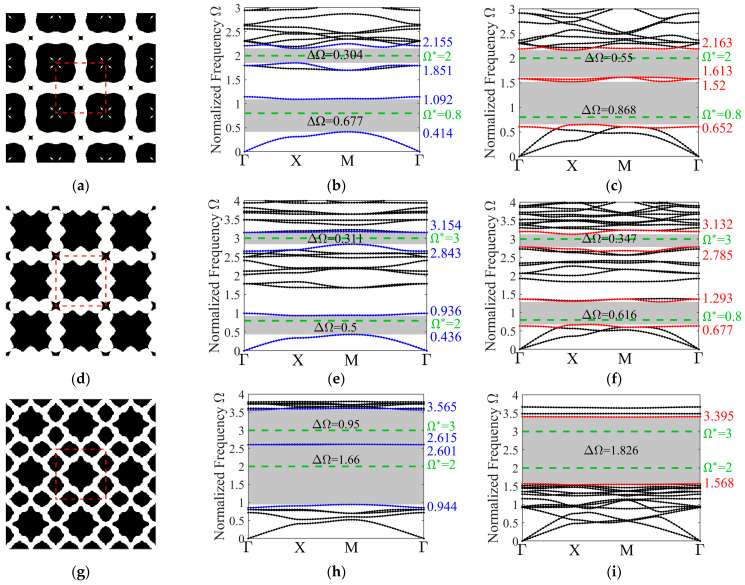
Characterization of the optimized 3 × 3 unit cell designs for various target frequency combinations, along with their corresponding band structures for out-of-plane and in-plane modes. The optimized configurations: (**a**) {0.8, 2}; (**d**) {0.8, 3}; (**g**) {2, 3}; the out-of-plane mode band structures: (**b**) {0.8, 2}; (**e**) {0.8, 3}; (**h**) {2, 3}; the in-plane mode band structures: (**c**) {0.8, 2}; (**f**) {0.8, 3}; (**i**) {2, 3}.

Finally, we present the optimized designs focusing on the maximization of complete band gaps for the dual-target frequencies. Figure 11 illustrates these optimized unit cells alongside their associated dispersion curves, encompassing both out-of-plane and in-plane wave modes. The target frequencies are indicated by green dashed lines within the band structure plots. The absolute widths of each band gap, along with their upper and lower bounds, are annotated in the corresponding figures. It is noteworthy that for the optimized design of target frequencies pair {2, 3}, the corresponding band structures exhibit two distinct band gaps in the out-of-plane mode, whereas only one merged band gap appears in the in-plane mode. This behavior differs from the optimization results obtained when the two modes are considered independently. Despite the escalated complexity of the optimization, the proposed strategy successfully realizes the optimized designs for dual-target complete band gaps, further confirming its efficacy and versatility.

## 4. Conclusions

A systematic topology optimization strategy to design the phononic crystal unit cells targeted at prescribed frequencies is proposed in this work. The primary goal is the tailoring of band gaps to ensure maximum bandwidths at specified target frequencies. Departing from traditional methods, the proposed objective function is formulated based on the signed minimal distance measure, which bypasses the common reliance on band orders and offers a more flexible design way. To tackle the complex optimization problem, we employ the KG-MFSE algorithm to pursue global optimization. This strategy is systematically applied to out-of-plane, in-plane, and complete wave modes. Comprehensive optimized designs are presented across a variety of target frequencies. These results validate the effectiveness of the proposed optimization strategy, revealing that the optimized unit cells exhibit remarkably wide band gaps at the specified target frequencies. The proposed strategy exhibits substantial potential for the control of elastic waves and the fabrication of devices with specific functional properties.

## Figures and Tables

**Table 1:** Comparison of related topology optimization strategies.

	Yi et al. [59]	Gómez-Silva et al. [60]	Wu et al. [61]	Present Work
Preset requirements	Target frequency and band gap orders	Target frequency and number of adjacent eigen frequencies	Target frequency	Target frequency
Optimization algorithm	Gradient-based	Gradient-based (BESO)	Gradient-based	Gradient-free (KG-MFSE)
Wave mode	In-plane	In-plane	In-plane	In-plane, out-of-plane, and complete
Number of target frequencies	Single	Single	Single	Single/dual

## Data Availability

The original contributions presented in this study are included in the article. Further inquiries can be directed to the corresponding author.
